# Novel β-Lactam/β-Lactamase Combination Versus Meropenem for Treating Nosocomial Pneumonia

**DOI:** 10.3390/antibiotics8040219

**Published:** 2019-11-13

**Authors:** Wei-Ting Lin, Chih-Cheng Lai, Chong-Un Cheong

**Affiliations:** 1 Department of Orthopedic, Chi Mei Medical Center, Tainan 71004, Taiwan; aapriliaa@gmail.com; 2 Department of Internal Medicine, Kaohsiung Veterans General Hospital, Tainan Branch 71051, Tainan, Taiwan; dtmed141@gmail.com; 3 Department of Intensive Care Medicine, Chi Mei Medical Center, Liouying 73657, Taiwan

**Keywords:** novel β-lactam/β-lactamase combinations, ceftolozane–tazobactam, ceftazidime–avibactam, nosocomial pneumonia, ventilator-associated pneumonia

## Abstract

This study reports the integrated analysis of two phase III studies of novel β-lactam/β-lactamase combination versus meropenem for treating nosocomial pneumonia (NP) including ventilator-associated pneumonia (VAP). The ASPECT-NP trial compared the efficacy and safety of ceftolozane–tazobactam versus meropenem for treating NP/VAP. The REPROVE trial compared ceftazidime–avibactam and meropenem in the treatment of NP/VAP. A total of 1528 patients (361 in the ceftolozane–tazobactam group; 405 in the ceftazidime–avibactam group; 762 in the meropenem group) were analyzed. The clinical cure rates at test-of-cure among the novel β-lactam/β-lactamase combinations group were non-inferior to those of the meropenem (70.7% vs. 72.1%, risk difference (RD) −0.01, 95% confidence interval (CI) 0.06–0.05) in the clinical evaluable populations. Overall 28-day mortality did not differ between novel β-lactam/β-lactamase combinations and the meropenem group (RD, −0.02, 95% CI, −0.09 to 0.05). Regarding the microbiological eradication rate, novel β-lactam/β-lactamase combinations were non-inferior to meropenem for *Pseudomonas aeruginosa*, *Klebsiella pneumoniae*, *Proteus mirabilis*, *Haemophilus influenzae*, *Staphylococcus marcescens*, and *Enterobacter cloacae*. Finally, novel β-lactam/β-lactamase combinations had a similar risk of (i) treatment-emergent adverse events (RD, 0.02, 95% CI, −0.02 to 0.06), (ii) events leading to the discontinuation of the study drug (RD, 0.00, 95% CI, −0.02 to 0.03), (iii) severe adverse events (RD, 0.03, 95% CI, −0.01 to 0.07), and (iv) death (RD, 0.02, 95% CI, −0.02 to 0.05) when compared with meropenem group. In conclusion, our findings suggest that novel β-lactam/β-lactamase combinations of ceftolozane−tazobactam and ceftazidime–avibactam can be recommended as one of the therapeutic options in the treatment of NP/VAP.

## 1. Introduction

Nosocomial pneumonia (NP), including ventilator-associated pneumonia (VAP), is the most common type of health care-associated infections [1]. Most importantly, NP/VAP can result in high morbidity and mortality. Nowadays, the emergence of antimicrobial resistance has largely limited the therapeutic option in this clinical condition. Therefore, a new antibiotic is urgently needed. Novel β-lactam/β-lactamase combinations including ceftazidime–avibactam and ceftolozane–tazobactam have demonstrated potent in vitro activity against many multidrug-resistant organisms, and both of them have demonstrated clinical efficacy in the treatment of complicated intra-abdominal infection (cIAI) and complicated urinary tract infection (cUTI) [2,3,4,5,6]. However, their role in the entity of NP/VAP remains unclear. Recently, two large randomized trials, ASPECT-NP [7] and REPROVE [8], investigated the clinical efficacy and safety of ceftolozane–tazobactam and ceftazidime–avibactam for treating NP, including VAP. To provide updated evidence regarding the usefulness of novel β-lactam/β-lactamase combinations in the treatment of NP/VAP, we performed an integrated analysis of these two studies [7,8].

## 2. Methods

From a literature search using Pubmed, only two randomized clinical trials [7,8] were found to investigate the clinical usefulness of novel β-lactam/β-lactamase combinations in the treatment of NP/VAP. Table 1 summarizes the characteristics of the two included studies. In the ASPECT-NP trial [7], Kollef et al. compared the efficacy and safety of ceftolozane-tazobactam (2 g ceftolozane and 1 g tazobactam every 8 hours) versus meropenem (1 g every 8 hours) for treating NP/VAP caused by gram-negative bacteria (GNB) over 8–14 days. In the REPROVE trial [8], Torres et al. compared ceftazidime–avibactam (2 g ceftazidime and 0.5 g avibactam every 8 hours) and meropenem (1 g every 8 hours) for 7–14 days in the treatment of NP/VAP. Both of them were multicenter, multinational, double-blind, phase-3 non-inferiority studies [7,8]. Statistical analyses were conducted by Review Manager version 5.3, using the random-effects model. The heterogeneity proportion was assessed using the *I^2^* measure. Heterogeneity was considered significant at *p* < 0.10 or I2 > 50%. The analyses of outcomes were calculated as pooled risk differences (RDs) and 95% confidence intervals (CIs). Only data obtainable in both studies were extracted for subgroup analysis.

## 3. Results

Overall, a total of 1528 patients (361 from the ceftolozane–tazobactam group; 405 from the ceftazidime–avibactam group; 762 from the meropenem group) were used in the analysis. Their mean age was 60.8 years, and 48.0% (*n* = 734) of patients were ≥65 years. The percentage of male patients was 73.2% (*n* = 1119) and 62.1% (*n* = 949) of patients were white. Overall, Enterobacteriaceae spp. contributed 72.1% (*n* = 639) of pathogens and *Klebsiella pneumoniae* (*n* = 307, 34.7%) was the most common pathogen, followed by *Pseudomonas aeruginosa* (*n* = 233, 26.3%), *Escherichia coli* (*n* = 130, 14.7%), *Enterobacter cloacae* spp. (*n* = 81, 9.1%), *Haemophilus influenzae* (*n* = 79, 8.9%), *Proteus mirabilis* (*n* = 70, 8.0%), *Serratia marcescens* (*n* = 58, 6.5%), *Staphylococcus aureus* (*n* = 58, 6.5%), and *Acinetobacter baumannii* (*n* = 38, 4.3%).

**Figure 1:**
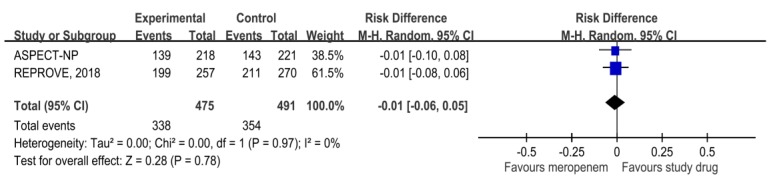
Clinical cure rate at test of cure for patients with nosocomial pneumonia among the novel β-lactam/β-lactamase combinations and meropenem groups.

The clinical cure rates at test-of-cure among the novel β-lactam/β-lactamase combinations group were non-inferior to those of the meropenem (70.7% vs. 72.1%, RD, −0.01, 95% CI, 0.06−0.05) in the clinical evaluable populations (Figure 1). In addition, the overall 28-day mortality did not differ between novel β-lactam/β-lactamase combinations and the meropenem group (RD, −0.02, 95% CI, −0.09 to 0.05). For patients with VAP, no significant difference was observed between novel β-lactam/β-lactamase combinations and the meropenem group (RD, −0.00, 95% CI, −0.07 to 0.07). In terms of microbiological eradication rate, novel β-lactam/β-lactamase combinations were non-inferior to meropenem for *P. aeruginosa* (RD, 0.14, 95% CI, −0.07 to 0.34), *E. coli* (RD, 0.03, 95% CI, −0.13 to 0.20), *K. pneumoniae* (RD, 0.07, 95% CI, −0.06 to 0.20), *P. mirabilis* (RD, 0.07, 95% CI, −0.31 to 0.44), *H. influenzae* (RD, 0.22, 95% CI, −0.16 to 0.59), *S. marcescens* (RD, 0.05, 95% CI, −0.29 to 0.39), and *E. cloacae* (RD, 0.06, 95% CI, −0.32 to 0.44). Finally, novel β-lactam/β-lactamase combinations had a similar risk of (i) treatment-emergent adverse events (TEAEs) (RD, 0.02, 95% CI, −0.02 to 0.06), (ii) events leading to discontinuation of the study drug (RD, 0.00, 95% CI, −0.02 to 0.03), (iii) severe adverse events (AEs) (RD, 0.03, 95% CI, −0.01 to 0.07), and (iv) death (RD, 0.02, 95% CI, −0.02 to 0.05), when compared with meropenem group.

## 4. Discussion

In this study, we demonstrated that novel β-lactam/β-lactamase combinations were noninferior to meropenem in the treatment of NP/VAP. This is supported by the following evidence: Novel β-lactam/β-lactamase combinations were associated with a noninferior clinical cure rate, 28-day mortality, and microbiological eradication rate when compared with meropenem. The clinical efficacy of novel β-lactam/β-lactamase combinations can be also explained by their potent in vitro activities. In the REPROVE trial [8], the minimum inhibitory concentrations of ceftazidime–avibactam required to inhibit the growth of 90% of organisms (MIC_90_) were only 0.5 mg/L and 8 mg/L against Enterobacteriaceae (*n* = 317) and *P. aeruginosa* (*n* = 101) isolates, respectively. This is consistent with International Network for Optimal Resistance Monitoring (INFORM) global surveillance investigations [9,10,11] that 99.0% to 99.4% of more than 30,000 Enterobacteriaceae isolates and 88.7–93.2% of 7062 clinical isolates of *P. aeruginosa* were susceptible to ceftazidime–avibactam. In the ASPECT-NP trial [7], 58 (13%) of 456 Enterobacteriaceae isolates and four (3%) of the 127 *P. aeruginosa* isolates were resistant to ceftolozane–tazobactam. The overall MICs of ceftolozane–tazobactam required to inhibit the growth of 50% of organisms (MIC_50_) were only 0.5 and 16 mg/L, respectively. This is in line with previous studies [12,13] that established the MIC_50_/MIC_90_ values of ceftolozane–tazobactam against *E. coli*, *K. pneumoniae*, and *P. aeruginosa* isolates in Germany and Spain as 0.25/0.5, 0.25/1–4, and 0.5/2–4 mg/L, respectively. All these findings based on the clinical and microbiological aspect should support the potential role of novel β-lactam/β-lactamase combinations in treating NP/VAP caused by GNB.

In addition, safety is an important concern regarding the use of novel antibiotics. In this study, we demonstrated that novel β-lactam/β-lactamase combinations were as tolerable as meropenem in many ways, including TEAE, discontinuation of study drug due to AE, severe AE, and death. This is consistent with a previous analysis of novel β-lactam/β-lactamase combinations in the treatment of cIAI and cUTI [2].

This study has several limitations. First, only two studies were included and the number of patients was limited. Second, some data were not available, and therefore more subgroup analysis, such as for elderly patients and multidrug-resistant organism populations, could not be performed.

In conclusion, the clinical efficacy, microbiological eradication, and safety profiles of novel β-lactam/β-lactamase combinations for treating NP/VAP were noninferior to meropenem. This suggests that novel β-lactam/β-lactamase combinations of ceftolozane–tazobactam and ceftazidime–avibactam can be recommended as one of the therapeutic options in the treatment of NP/VAP. In contrast to previous studies investigating individual novel β-lactam/β-lactamase combinations, this study was an integrated analysis of two antibiotics in the same class. Although both ceftolozane–tazobactam and ceftazidime–avibactam have been classified as novel β-lactam/β-lactamase combinations, these two agents are not the same in many ways. Further study is warranted to investigate the usefulness of each agent for treating NP/VAP.

## Figures and Tables

**Table 1:** Characteristics of the Studies.

Study	Study Design	Study Duration	Study Site	Study Population	Regimen	
					Novel β-Lactam/β-Lactamase Combinations	Meropenem
REPROVE, 2018	prospective, parallel-group, randomized, double-blind, double-dummy, phase 3 non-inferiority trial	2013–2015	136 hospitals in 23 countries	Adult patients in hospital, and had NP	2 g ceftazidime and 0.5 g avibactam intravenous infusion every 8 h.	1 g meropenem intravenous infusions every 8 h
ASPECT-NP, 2019	randomized, controlled, double-blind, phase 3, non-inferiority trial	201–-2018	263 hospitals in 34 countries	Adult patients, were intubated and requiring MV, and had VAP or ventilated NP caused by Gram-negative bacteria	2 g ceftolozane and 1 g tazobactam intravenous infusions every 8 h	1 g meropenem intravenous infusions every 8 h

NP, nosocomial pneumonia; VAP, ventilator-associated pneumonia; MV, mechanical ventilation.
